# Chemical and Structural Characterization of Several Mid-Term Explanted Breast Prostheses

**DOI:** 10.3390/ma9080678

**Published:** 2016-08-09

**Authors:** Angela Amoresano, Luca De Stefano, Ilaria Rea, Federica Pane, Leila Birolo, Fabrizio Schonauer

**Affiliations:** 1 Dipartimento di Scienze Chimiche, Complesso Universitario di Monte Sant’Angelo, Via Cinthia, Napoli 80100, Italy; angela.amoresano@unina.it (A.A.); federica.pane@unina.it (F.P.); leila.birolo@cnr.it (L.B.); 2 CNR-IMM, Istituto per la Microelettronica e Microsistemi, Unità di Napoli, Via P. Castellino 111, Napoli 80131, Italy; ilaria.rea@cnr.it; 3 Dipartimento di Patologia Sistematica, University of Napoli “Federico II”, Via S. Pansini 5, Napoli 80131, Italy; fabrizio.schonauer@unina.it

**Keywords:** breast implants, organic and inorganic contamination, materials science

## Abstract

The recent scandal of poly implant prostheses (PIP), which were found in some cases to be made of non-medical grade silicone (as reported by the European Scientific Committee on Emerging and Newly Identified Health Risks), had a great social impact. Thousands of patients asked for implant removal with significant costs for public health care systems. We analysed, by a multidisciplinary approach, sixteen different breast implants after explantation by using several analytical and structural techniques, such as Fourier Transform infrared spectroscopy (FT-IR), mass spectrometry equipped by ion coupled plasma (ICP-MS), gas-chromatography (GC-MS), and tensile testing. Traces of organic (fatty acid) and inorganic (Fe, Cr, Pt, Na, and other metals) substances were found in all samples, and, even if these values are under danger threshold levels, our study results highlight the possibility of bioaccumulation and tissue contamination, implying the need for continuous medical surveillance and monitoring of material aging.

## 1. Introduction

Silicone based breast implants have been used for more than 40 years in reconstructive and aesthetic surgery due to high biocompatibility and tolerance. These prostheses are made of polymeric shells, with textured or smooth surfaces, filled by silicone gel, usually of high purity (medical grade) polydimethylsiloxane (PDMS). Materials science studies tried to solve three main criticisms of these materials: capsular contracture, gel bleeding, and shell rupture, which are mainly due to the changing mechanical properties of the gel; in particular, different mixtures of less viscous and cohesive gels which are used in their fabrication. The recent case of poly implant prostheses (PIP) has highlighted that, beyond the aging materials’ problem, the mechanics of which are not clearly understood, material composition is also a “hot topic” that should be addressed by all manufacturers [1,2]. Evidence of PIP rupture rate almost doubled compared to other implants, and have forced several national health agencies to investigate PIP implant characteristics. A correlation between prosthesis aging, rupture, and infiltration of human body components into the prosthesis’ shell and silicone gel has been described in the past [3,4,5,6,7]. Some studies on long-term implantation confirmed lipids absorption with consequent polymer swelling and consequential prosthesis rupture [8,9,10,11]. The Scientific Committee on Emerging and Newly Identified Health Risks (SCENIHR) has recently published an upgrade to its guidelines on the safety of PIP silicone breast implants [12]. Although the Committee did not recognise any direct relationship between rupture events and breast cancer risk, making routinely mass removal of intact PIP implants non-justifiable, breast implant–associated anaplastic large-cell lymphoma (ALCL) has been recently described as a clinicopathologic entity that usually presents as an effusion-associated fibrous capsule surrounding an implant [13,14,15,16]. A link between the textured surface implants and ALCL has also been highlighted. Based upon these important findings, every single adverse implant case must be judged from a physician and surgeon’s point of view. Following the investigation scheme as proposed by SCENIHR, we analysed some recently implanted prostheses, as well never implanted ones, searching for organic and inorganic contents, using different analytical techniques. We applied materials science characterization approach included Fourier Transform infrared spectroscopy (FT-IR), tensile testing, and analytical chemistry methods, as mass spectrometry equipped by ion coupled plasma (ICP-MS) and gas-chromatography (GC-MS), in order to fully characterize explanted breast implants.

## 2. Results

### 2.1. Naked Eye and Photographic Images

**Figure 1:**
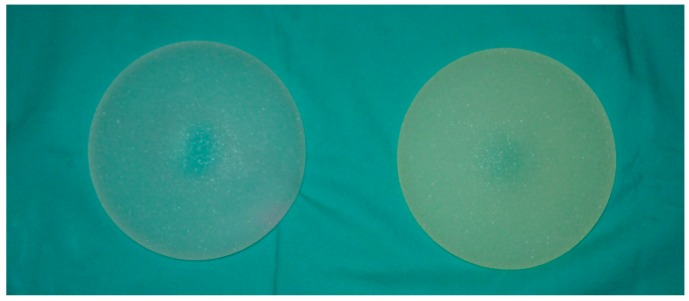
Digital images of breast prostheses, for example. On the left, a never-implanted sample having a white translucent colour; on the right, a six-month explanted sample with a dark yellow colour.

In Figure 1 digital images of two prostheses are reported for comparison and as an example of real samples used in our study. The sample on the left had never be implanted, and was white and partially translucent; the six-months explanted sample on the right revealed a yellowish, uniform cover due to human organic matter adsorbed on the outer shell of the prosthesis. Other older samples (i.e., two-year explanted samples) were more brownish, most likely due to stratification of biological matter.

### 2.2. FT-IR Characterization

**Figure 2:**
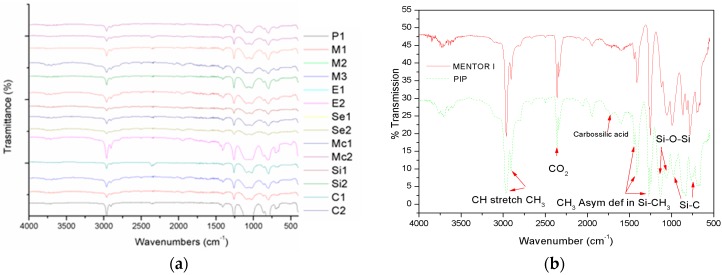
(**a**) FT-IR spectra of silicone gels extracted from all samples; (**b**) FT-IR analysis of silicone gel extracted from PIP I (PIP II completely overlaps to PIP I) and a never-implanted prosthesis, as a reference of “pure” PDMS material.

In Figure 2a, we report the FTIR spectra of all our samples, with the exception of the two never implanted: all of the spectra were very similar and really close to a pure PDMS spectrum, as can be found in the literature [17]. A careful analysis of each trace revealed interesting features in case of PIP I and PIP II samples (see Figure 2b): even if FTIR intrinsic sensitivity prevented trace elements monitoring, a carboxylic acid characteristic peak (1710 cm^−1^) can be highlighted in both PIP I and II (not shown in Figure 2b due to overlap) spectra. This was a clear indication that PIP silicone included fatty acid coming from the human body. In all other spectra, this peak was absent.

### 2.3. ICP-MS Analysis

The multi-elemental analysis was performed by ion-coupled plasma mass spectrometry (ICP-MS). The standard addition approach for calibration on three concentration levels was used in order to keep the matrix induced variations under control. A minimum of three replicates of each calibration standard was run. Intra-day repeatability was determined by the measurements of a sample three times on the same day. Inter-day repeatability was determined by the analysis of a similar digested solution on three different days over a period of one week. Relative standard deviation (RSD) was calculated for both series of analyses. Reagent blanks run together with matrices. Metals detected and quantified are shown in Table 1a (Li, Mn, Fe, Co, Mo, Sb, Pt) and 1b (B, Si, P, Cr, Fe, Ni, Zn, Ge), where the detection limit for each metal is indicated when found. ICP-MS analysis let us to identify and quantify up to 53 different metal ions in a single run. Among these, only 15 resulted in having a value higher than the ICP limit of detection, thus contributing to the elemental composition of the analysed samples. The high sensitivity (ppt) of ICPMS analysis led to the detection and simultaneous quantization of trace elements, such as Se, Sb, and In.

The mass spectral analyses demonstrated that the metal composition in both explanted and never-implanted samples was quite similar, even if some differences could be appreciated both qualitatively and in quantitatively. However, concentrations of some elements were completely different, with some present in one sample, while completely absent in others. This could be due to the intrinsic heterogeneity of the prosthesis samples. The presence of some metals was not clear at all: if Si and Pt could be related to the manufacturing processes, the others were not. In particular, Cr, Ni, and Zn, quite homogeneously present in all samples, were not normally used as catalysts in silicone synthesis, so that their provenience could not be explained.

### 2.4. GC-MS Analysis

The organic composition of different prosthesis was determined by GC-MS analyses after liquid-liquid extraction of different analytes from silicone moiety. Aliquots of the extracted mixtures from different samples were derivatized to TMSA-derivatives and directly analysed by GC-MS by monitoring the total ion current (TIC) as a function of time. Each species was univocally identified on the basis of the electron impact fragmentation spectra. All of the analyses were performed as triplicates. The TIC chromatograms revealed the presence of a large number of species. Among these, the major components were identified as cyclic siloxane components. However, differences in siloxane composition can be appreciated both in qualitative and in quantitative terms, as shown in Table 2 which reports the attribution of each species and the corresponding amount (as a percentage). Our procedure provided the identification of other several minor species in the different silicone preparations and results are summarised in Table 2a–c). As shown in Table 2a, other species than cyclic (2b) and linear (2c) siloxane can be observed belonging to different organic components. In particular, linear hydrocarbons and fatty acids can be detected in some samples, even if always in very low concentration. This is not surprising since both outer shell silicone and standard PDMS could be easily permeable to lipid molecules. As expected, Mentor IV did not contain these substances.

### 2.5. Proteomic Analysis

Proteomic analyses were carried out in order to detect any possible infiltration of protein material inside the breast implants. Explanted implants from several companies were analysed for the presence of peptides/proteins on the inner part of the implant shell. In any case, regardless of the manufacturer, proteomic analysis could not detect the presence of any peptide that could be ascribed to the presence of proteinaceous material inside, or protruding from, the inner side of the implant shell. This result excluded, at least in the samples studied in our work, protein infiltration through the external jacket of implants.

### 2.6. Mechanical Characterization

Results of mechanical characterizations are reported in Table 3: Young’s modulus, strain at break, and stress at break, measured by a tensile strength tester following a standard procedure, are quantified for each sample. Values of Young’s modulus were almost all close to 1 MPa, quite common for plastic materials [18], except for PIP 2 and Cox 2 samples, which were significantly lower. In the case of strain at break, which is the relative elongation of the sample at break and expresses the capability of a material to resist changes of shape without crack formation, the variability was much higher even among samples of the same manufacturer. The same observation can be made for the tensile strength at break, which is the tensile stress at the moment at which a test specimen tears, and which, in our experiment, ranged from 1.08 to 8.75 MPa in value.

## 3. Discussion

Regardless the kind of texture (i.e. fine or coarse) of the outer prosthesis shells, when explanted from the body, all samples were completely covered by biological residues that altered the colour of the device and could be not removed, even by extensive rinsing in physiological solution. The accumulation of cells, lipids, plasma, and blood on the outer shell was a clear indication of material aging, but it was difficult to quantify the aging effect, as we could not compare the never-implanted samples with the explanted ones for each producer. We were also interested in assessing the transport of biological/organic matter through the outer shell in the gel material inside the implants. Infrared spectrometry confirmed the presence of such substances in some samples: FTIR analysis revealed that all implants were made of almost identical material, i.e., PDMS, since our samples’ spectra were very similar, and showed signs of carboxylic acid only in PIP prostheses that could be ascribed to lipid infiltration. Traces of organics and metals were highlighted by ICP-MS and GS-MS spectroscopies. It was concluded that the origin of organic impurities could not surely addressed to mass transfer through the implants’ outer shell, while all other impurities could be related to the fabrication process. Proteomic analysis did not detect any protein or peptide in the gel sampled. By examining average values of content for each substance, no direct correlation between their amount and the mechanical behaviour of the outer shell was observed. This is most likely due to the small number of samples included in our study. It was noted that all explanted outer shell samples underwent greater deformations than those registered from the non-implanted prosthesis, thereby revealing an almost obvious effect of mechanical weakening caused by implant aging. In particular, PIP and Cox prosthesis revealed the greatest weakening compared to all other samples. PIP and Cox implants showed breaking points at the lowest strain value with respect to all others, which again was further indication of poor mechanical properties. We were not able to determine the causes of the mechanical property deterioration, but can hypothesize that these could be ascribed to both production (quality of materials and/or manufacturing techniques) and more rapid aging of the prosthesis during the period of use.

## 4. Materials and Methods 

### 4.1. Breast Implant Samples 

We collected 16 prosthesis, all explanted over a period between one and two years. Implants were by different manufactures from all over the world, in particular, four by Mentor (USA) (Mentor 1 < 1 year; Mentor 2 > 1 year), two by PIP (France) (PIP 1 < 1 year; PIP 2 > 1 year), McGhan (USA) (Mc 1 < 1 year; Mc 2 > 1 year), Eurosilicone (France) (E 1 < 1 year; E 2 > 1 year), Sebin Laboratoire (France) (Se 1 < 1 year; Se 2 > 1 year), Silimed (Brasil) (Si 1 < 1 year; Si 2 > 1 year), and Cox-Uphoff International (USA) (C 1 < 1 year; C 2 > 1 year). Despite the period of implantation, all prostheses had the external, hard silicon shell completely covered by organic material. Colour ranged from yellowish or slightly brown in all cases, far from their original rough white colour at the time of implant and in comparison to the non-implanted ones (see Figure 1). Mentor 3 and 4 were control samples, since they had never been implanted. While Mentor 3 had been stored with other explanted prostheses without any protection, Mentor 4 was still sealed in its original package at analysis time. All implants, explanted and the two never implanted, had textured surface, and had been washed in antiseptic solution for sterilization and in physiological solution before storage.

### 4.2. FT-IR Characterization

We have characterized the silicone gel of each implant by FT-IR spectroscopy (Thermo Scientific-Nicholet Continuµm XL, USA): spectra were acquired 32 times between 400 and 4000 cm^−1^ with a resolution of 4 cm^−1^. The inner material was collected by a plastic needle using a syringe after a longitudinal cut in the implant shell of a few centimetres, in order to carefully avoid external contaminations.

### 4.3. ICP-MS Analysis

An amount of 5 g for each sample was suspended in 50 mL water for 72 h at room temperature. Aliquots of water solution from each sample were directly analysed by ICP-MS (Agilent, USA). The solution was then transferred into polystyrene liners, an aliquot of each sample was diluted 1:10 v/v with 5% HNO_3_ and finally analysed with an Agilent 7700 ICP-MS from Agilent Technologies, equipped with a frequency-matching RF generator and third-generation octopole reaction system (ORS3), operating with helium gas in ORF. The following parameters were used: radiofrequency power 1550 W; plasma gas flow 14 L/min; carrier gas flow 0.99 L/min; He gas flow 4.3 mL/min. ^103^Rh was used as an internal standard (50 μg/L final concentration). Multi-element calibration standards were prepared in 5% HNO_3_ at four different concentrations (1; 10; 50; and 100 μg/L).

### 4.4. GC-MS Analysis

Aliquots of each prosthesis were submitted to a liquid-liquid extraction procedure by using an equal amount of chloroform (1:1 v/v). The extraction step was performed three times and the organic substances were collected and dried. Analyte mixtures were finally trimethylsilylated in 200 μL of N, *O*-bis(trimethylsilyl) acetamide (TMSA) at 80 °C for 45 min. The sample was dried down under nitrogen, dissolved in 200 μL of hexane and centrifuged to remove the excess of solid reagents. The hexane supernatant (1/200) was used for the GC-MS analysis. GC-MS analyses were performed on a 5390 MSD quadrupole mass spectrometer (Agilent Technologies, USA) equipped with a gas chromatograph by using a SPB-5 fused silica capillary column (30 m, 0.5 mm ID, 0.25 μm ft) from Supelco. The injection temperature was 250 °C. The oven temperature was increased from 40 °C to 90 °C in 1 min and held at 90 °C for 1 min before increasing to 140 °C at 25 °C/min, to 200 °C at 5 °C/min, and finally to 300 °C at 10 °C/min. Electron ionisation (EI) mass spectra were recorded by continuous quadrupole scanning at 70 eV ionisation energy.

### 4.5. Proteomic Analyses

**Figure 3:**
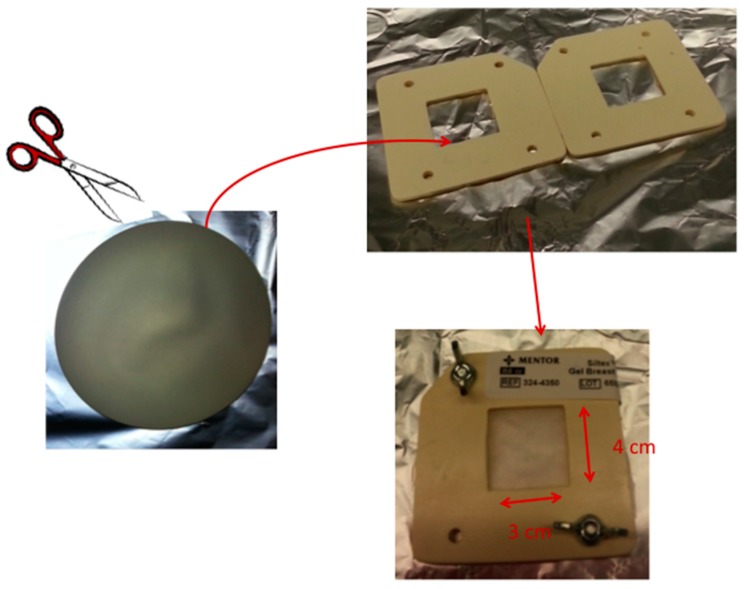
Scheme of sample preparation for proteomics analysis.

Search of proteinaceous material was carried out following a general proteomic analytical procedure based on identification of peptides by liquid chromatography-tandem mass spectroscopy (LC-MS/MS) after an enzymatic reaction in heterogeneous phase by proteomics-grade trypsin, as previously described [19]. A mechanical frame was realized (see scheme reported in Figure 3) to properly allocate a section 4 cm × 3 cm of implant outer shell, in order to locally perform the enzymatic reaction on the inner surface of the implant shell. For each sample, 100 μL of 10 ng/μL proteomics-grade trypsin in 10 mM ammonium bicarbonate pH 7.5 was added on the outer shell surface and incubated at 37 °C for 16 h. The supernatants were then recovered, 0.22 μm filtered, dried in Speed-Vac and suspended again in 20 μL of 0.1% formic acid in Milli-Q water. The samples were analysed using a CHIP MS 6520 QTOF mass spectrometer equipped with a capillary 1200 HPLC system and a chip cube (Agilent Technologies, Palo Alto, CA, USA). After loading, the sample was first concentrated and washed at 4 µL·min^-1^ in 40 nL enrichment column (Agilent Technologies chip), with 0.2% formic acid in 2% acetonitrile as eluent. The sample was then fractionated on a C18 reverse-phase capillary column (75 μm × 43 mm in the Agilent Technologies chip) at a flow rate of 400 nL·min^−1^ with a linear gradient of eluent B (0.2% formic acid in 95% acetonitrile) in A (0.2% formic acid in 2% acetonitrile) from 7% to 60% in 50 min. Raw LC–MS/MS data files were acquired using data-dependent acquisition of one MS scan (mass range from 400 to 2000 m/z) followed by MS/MS scans of the three most abundant ions in each MS scan. Raw data from nano-LC–MS/MS analyses were used to query protein databases (NCBI, with the taxonomy restriction to *Homo sapiens*) using in house MASCOT software (Matrix Science, Boston, MA, USA). Peptide modification settings were: fixed modification, carbamidomethylation on Cystein; variable modifications, oxidation on Methylation, and the possible formation of pyroglutammic acid from glutamine residues at the N-terminal position of peptides. 

### 4.6. Mechanical Characterization

Samples of the outer shell of implants have been examined following the standard procedure reported in UNI EN ISO 104301-2012 standard test method for tensile properties of plastics. Briefly, three to five samples for each implant has been cut into a dog-bone shape, 50 mm long, from the outer silicon shell of prostheses and measurements between 0 and 4 MPa were performed using a tensile tester (Instrom, model 5569, USA).

## 5. Conclusions

From images recorded of explanted prostheses it was well evident that macroscopic changes to the outer shell of the aged implants could be observed: from a three-month aged implant up to two-year aged one, the biological matter had continuously transferred from body to implant, covering it completely. The change of colour was not considered significant with respect to rupture events and deeper analyses were required.

Even if all implants were medium term explanted (1–2 years), traces of organic substances, not directly related to silicone production process, were found in some analysed samples. Their origins come from both contamination of the silicone gels during production processes, or infiltration from human bodies. In the latter, we expected a gradient of concentrations from the outer surface to the inner surface of the implant, which, however, we were unable to detect. While it was true that the absolute values of species concentration were far from being considered dangerous to human health, we found it very difficult to estimate the impact on the materials’ aging. The same observation could be done for metals. Due to the PDMS production process, all samples contain metals, such as Si and Pt [19], but of the others found, such as Cr, Zn, and Ni, whose origin was questionable, and even if the fact that their quantities were below the danger thresholds for human health, we were unable to exclude them having a negative impact on implant life. Based upon the results of our characterization approach, we can exclude protein infiltration in the inner material of breast implants. It is doubtful that proteins eventually channelled through the external shell of the implant were so strongly bound to silicone that we did not reveal this with our proteomic analysis. Our study has led us to confirm that the presence of organic pollutants and toxic metals which, as revealed by our analysis, are found in medical-grade materials used in breast implants, strongly suggests strict monitoring of their bioaccumulation and further study of their consequences on the human body.

## Figures and Tables

**Table:** (**a**)

Breast Implant	Li (μg/L)	Mn (μg/L)	Fe (μg/L)	Co (μg/L)	Mo (μg/L)	Sb (μg/L)	Pt (μg/L)
Mentor 1	<1.0	2.9 ± 0.3	15 ± 5	0.67 ± 0.06	0.6 ± 0.3	1.70 ± 0.1	9 ± 4
Mentor 2	<1.0	2.1 ± 0.8	<0.716	0.55 ± 0.10	0.53 ± 0.04	1.55 ± 0.03	55 ± 7
Mentor 3	3 ± 1	2.1 ± 0.7	<0.716	0.63 ± 0.02	0.56 ± 0.02	1.62 ± 0.15	23 ± 10
Mentor 4	<1.0	2.7 ± 0.7	4 ± 2	0.66 ± 0.06	0.57 ± 0.14	1.88 ± 0.13	4.7 ± 0.8
Pip 1	5 ± 3	2.6 ± 0.6	11 ± 7	0.64 ± 0.07	0.54 ± 0.09	1.78 ± 0.16	0.8 ± 0.3
Pip 2	6.6 ± 0.5	1.9 ± 0.3	<0.716	0.55 ± 0.17	0.64 ± 0.12	1.48 ± 0.04	1.2 ± 0.2
Mcghan 1	6 ± 3	1.6 ± 0.2	<0.716	0.51 ± 0.15	0.30 ± 0.02	1.52 ± 0.16	50 ± 9
Mcghan 2	5 ± 3	2.9 ± 0.3	8 ± 4	0.67 ± 0.01	0.55 ± 0.16	1.9 ± 0.3	22 ± 8
Eurosilicon 1	9.8 ± 0.1	2.1 ± 0.4	<0.716	0.53 ± 0.11	0.59 ± 0.16	1.5 ± 0.2	32 ± 5
Eurosilicon 2	5.8 ± 0.1	2.6 ± 0.4	<0.716	0.83 ± 0.18	0.51 ± 0.10	1.6 ± 0.2	21 ± 1
Silimed 1	6 ± 3	1.7 ± 0.5	<0.716	0.68 ± 0.08	0.55 ± 0.05	1.54 ± 0.19	1.4 ± 0.2
Silimed 2	7.1 ± 0.3	2.7 ± 0.5	<0.716	0.75 ± 0.04	0.50 ± 0.02	1.61 ± 0.11	2.4 ± 0.4
Sebin 1	14 ± 9	2.7 ± 0.8	<0.716	0.54 ± 0.03	0.5 ± 0.2	1.9 ± 0.2	<0.406
Sebin 2	8.7 ± 0.1	3.1 ± 0.3	<0.716	0.44 ± 0.10	0.69 ± 0.13	1.5 ± 0.2	27 ± 9
Cox 1	2.3 ± 0.1	2.4 ± 0.7	<0.716	0.62 ± 0.06	0.6 ± 0.2	1.63 ± 0.12	1.3 ± 0.2
Cox 2	<1.0	1.6 ± 0.2	<0.716	0.61 ± 0.09	0.43 ± 0.11	1.2 ± 0.2	3.3 ± 0.2
AVG	6.6	2.3	9.5	0.62	0.54	1.6	17

**Table:** (**b**)

Breast Implant	B (μg/L)	Si (μg/L)	P (μg/L)	Cr (μg/L)	Fe (μg/L)	Ni (μg/L)	Zn (μg/L)	Ge (μg/L)
Mentor 1	41 ± 10	441 ± 23	31 ± 2	97 ± 3	15 ± 5	45 ± 1	46 ± 2	11 ± 6
Mentor 2	0.95 ± 0.50	397 ± 13	27 ± 5	85 ± 3	<0.716	38.7 ± 0.7	47 ± 2	6.0 ± 0.9
Mentor 3	<0.5	285 ± 8	31 ± 1	86.0 ± 0.9	<0.716	37.6 ± 1.7	45.7 ± 0.9	<0.652
Mentor 4	<0.5	238 ± 8	27 ± 4	93 ± 2	4 ± 2	43.4 ± 1.4	46.1 ± 0.8	<0.652
Pip 1	19 ± 9	539 ± 10	31 ± 7	96 ± 3	11 ± 7	42.8 ± 1.5	48 ± 2	18 ± 6
Pip 2	23 ± 6	529 ± 8	25 ± 4	85 ± 5	<0.716	38 ± 2	48.5 ± 0.7	28 ± 5
Mcghan 1	<0.5	319 ± 7	27 ± 4	80 ± 2	<0.716	37.2 ± 1.0	45 ± 2	<0.652
Mcghan 2	<0.5	294 ± 2	35 ± 2	94 ± 2	8 ± 4	40.4 ± 1.7	46.4 ± 1.2	<0.652
Eurosilicon 1	<0.5	280 ± 3	24 ± 3	82 ± 4	<0.716	37.1 ± 1.7	45 ± 2	<0.652
Eurosilicon 2	<0.5	298 ± 3	21 ± 3	88 ± 4	<0.716	43.1 ± 1.1	49 ± 2	<0.652
Silimed 1	<0.5	208 ± 4	28 ± 5	85 ± 1	<0.716	36.9 ± 1.1	43.6 ± 1.3	<0.652
Silimed 2	<0.5	267 ± 5	19 ± 3	91 ± 2	<0.716	35.1 ± 0.7	41 ± 1	<0.652
Sebin 1	<0.5	346 ± 4	24 ± 4	89 ± 3	<0.716	40 ± 2	47 ± 2	<0.652
Sebin 2	<0.5	406 ± 2	32 ± 1	82 ± 4	<0.716	44 ± 1	49 ± 3	<0.652
Cox 1	<0.5	209 ± 1	31 ± 4	90.0 ± 0.4	<0.716	39 ± 2	44.5 ± 0.2	<0.652
Cox 2	<0.5	216 ± 5	25 ± 4	74 ± 2	<0.716	32.1 ± 0.8	42.9 ± 1.6	<0.652
AVG	21	329	27	87	9	39	46	16

**Table:** (**a**)

Organic Substances	M 1	M 2	Mc 1	Mc 2	E 1	C 1	P 2	S 1
1-Chlorodecane (%)	–	–	0.1	0.4	1.1	–	–	–
1-Chlorotetradecane (%)	–	–	–	–	0.2	–	–	
Octadecenoic Acid (%)	–	–	–	–	–	–	0.01	0.04
Palmitic acid (%)	0.5	–	–	–	–	–	–	–
Oleic acid (%)	–	0.1	–	–	–	–	0.1	–
Stearic acid (%)	–	–	–	–	–	0.3	1.7	0.1

**Table:** (**b**)

Cyclic Siloxanes	M1	M2	M3	M4	P1	P2	Mc1	Mc2	E1	E2	Si1	Si2	Se1	Se2	C1	C2	AVG
C6	20.2	50.8	50.6	–	–	53.5	47.5	11.8	15.2	14.5	–	–	–	–	–	–	33
C8	24.3	23.8	22.1	–	–	24.1	20.9	26.8	25.1	21.6	–	–	18.8	21.8	–	–	23
C10	9.6	6.9	6.4	–	12.3	7.3	6.1	12.5	11.3	14.1	–	–	19.2	12.2	–	–	11
C12	2.7	1.6	1.4	–	7.2	1.7	1.4	7.2	7.3	9.3	–	–	8.8	7.8	–	–	5
C14	1.5	0.6	0.5	6.9	3.4	0.6	0.6	1.6	1.5	1.8	3.1	4.2	2.8	3.8	7.5	2.5	2.7
C16	0.6	0.3	0.2	2.9	2.1	0.2	0.1	0.5	0.2	0.4	0.7	0.5	1.6	1.9	4.6	1.4	1.1
C18	0.6	–	–	2.1	1.4	–	0.1	–	–	–	0.2	0.6	0.9	0.6	2.4	1.2	1
C20	0.4	–	–	1.8	0.9	–	0.4	–	–	–	0.2	0.4	0.5	0.7	2.2	0.9	0.8
C22	0.4	–	–	0.5	0.7	–	–	–	–	–	0.1	0.2	0.2	0.8	1.7	0.6	0.6
C24	–	–	–	0.9	0.6	–	–	–	–	–	0.05	0.1	–	–	1.2	0.3	0.52
C26	–	–	–	0.5	0.5	–	–	–	–	–	0.1	0.3	–	–	0.8	0.03	0.37
C28	–	–	–	0.1	0.4	–	–	–	–	–	0.02	0.07	–	–	0.7	0.02	0.22
C30	–	–	–	0.5	0.3	–	–	–	–	–	–	–	–	–	0.4	0.01	0.3
C32	–	–	–	0.03	0.2	–	–	–	–	–	–	–	–	–	0.3	0.02	0.14
C34	–	–	–	–	0.1	–	–	–	–	–	–	–	–	–	–	–	0.1
C36	–	–	–	–	0.1	–	–	–	–	–	–	–	–	–	–	–	0.1
C38	–	–	–	–	0.1	–	–	–	–	–	–	–	–	–	–	–	0.1

**Table:** (**c**)

Linear Siloxanes	M1	M2	M3	M4	P1	P2	Mc1	Mc2	E1	E2	Si1	Si2	Se1	Se2	C1	C2	AVG
C10	2.0	1.3	1.4	–	–	0.8	1.9	2.2	1.9	1.7	–	–	–	–	–	–	1.7
C12	4.3	2.3	2.3	1.4	–	1.6	3.1	5.4	5.3	4.3	23.1	19.1	4.1	3.8	0.6	21.7	7.2
C14	5.3	2.5	2.8	2.3	7.3	1.9	3.4	3.3	3.1	5.1	22.6	20.3	6.7	9.7	5.1	20.9	7.7
C16	5.4	2.1	2.5	2.8	7.9	1.8	3.2	6.9	6.5	7.5	17.1	15.2	8.3	7.3	8.8	15.5	7.4
C18	5.3	1.7	2.2	2.5	7.4	1.6	2.7	5.9	5.6	6.5	11.3	10.4	7.6	5.6	11.7	10.1	6.1
C20	4.6	1.1	1.4	2.2	5.9	1.1	1.8	3.9	4.2	3.3	8.2	6.2	6.1	3.1	13.2	7.2	4.6
C22	3.2	0.6	0.8	1.4	4.3	0.6	1.0	2.4	2.7	1.7	5.6	6.4	4.1	5.1	11.3	4.9	3.6
C24	2.2	0.3	0.4	0.8	3.5	0.4	0.5	1.3	1.6	1.1	3.2	2.1	2.8	1.8	7.4	3.1	2.0
C26	1.5	0.2	0.2	0.4	3.2	0.2	0.3	0.7	0.9	0.7	1.5	2.5	2.1	2.6	4.1	1.6	1.4
C28	0.8	–	0.1	0.2	2.7	0.1	0.2	0.4	0.5	0.5	0.6	1.6	1.1	1.9	2.1	0.7	0.9
C30	0.4	–	–	0.1	2.2	–	0.1	0.2	0.3	0.3	0.2	1.2	0.6	1.6	1.1	0.2	0.6
C32	–	–	–	1.4	1.6	–	–	–	–	–	0.2	0.4	–	–	0.7	0.2	0.7
C34	–	–	–	2.3	1.3	–	–	–	–	–	0.05	0.05	–	–	0.5	0.04	0.7
C36	–	–	–	2.8	0.9	–	–	–	–	–	–	–	–	–	0.6	0.1	1.1
C38	–	–	–	–	0.7	–	–	–	–	–	–	–	–	–	–	–	0.7
C40	–	–	–	–	0.6	–	–	–	–	–	–	–	–	–	–	–	0.6
C42	–	–	–	–	0.5	–	–	–	–	–	–	–	–	–	–	–	0.5
C44	–	–	–	–	0.4	–	–	–	–	–	–	–	–	–	–	–	0.4
C46	–	–	–	–	0.3	–	–	–	–	–	–	–	–	–	–	–	0.3

**Table 3:** Results of mechanical tests for all samples: Young’s modulus; stress at break; strain at break.

Breast Sample	MODULUS (MPa)	STRAIN AT BREAK (%)	STRESS AT BREAK (MPa)
Mentor 1	1.07	465	2.7
Mentor 2	1.26	686	7.3
Mentor 3	1.15	545	4.5
Mentor 4	1.37	776	8.75
Pip 1	0.94	282	2.08
Pip 2	0.7	204	1.6
Mcghan 1	1.12	510	5.12
Mcghan 2	0.97	347	2.8
Eurosilicon 1	0.77	292	1.97
Eurosilicon 2	1.03	313	4.2
Silimed 1	0.94	531	4.41
Silimed 2	1.36	404	3.6
Sebin 1	0.95	320	2.45
Sebin 2	1.12	431	3.18
Cox 1	1.06	435	2.18
Cox 2	0.86	395	1.08
AVG	1.04	433	3.6
